# An exploratory investigation of changes in gait parameters with age in elderly Japanese women

**DOI:** 10.1186/s40064-016-2739-7

**Published:** 2016-07-13

**Authors:** Irma Nur Afiah, Hiroki Nakashima, Ping Yeap Loh, Satoshi Muraki

**Affiliations:** Department of Human Science, Graduate School of Design, Kyushu University, Fukuoka, Japan; Research Fellow of Japan Society for the Promotion of Science, Tokyo, Japan; Department of Human Science, Faculty of Design, Kyushu University, Fukuoka, Japan

**Keywords:** Aging, Walking motion, Elderly Japanese women, Gait characteristics

## Abstract

**Background:**

The aim of the present study was to identify gait parameters in elderly Japanese women. 30 elderly women (65–74.9 years old) and 19 very elderly women (≥75 years old) participated in this study. A 3-dimensional (3D) motion analysis system was used to collect kinematic data, and a total of 70 gait parameters were analysed. Gait parameters included basic gait parameters, gait cycle parameters, and joints angle parameters, as well as angular velocity parameters, such as peak velocity and timing at the hip, knee, and ankle joints.

**Results:**

Our results indicated that basic gait parameters, such as the gait cycle, peak joint angle timing, and angular velocity parameters, significantly differ between elderly and very elderly women. Delayed peak joint angle timing and angular velocity parameters occurred during critical phases throughout the gait cycle: pre-swing, initial swing, and terminal swing phases.

**Conclusions:**

Several gait parameters exhibited significant differences between elderly and very elderly women. The timing of the peak joint angle and angular velocity parameters are primary characteristics defining gait changes in the elderly.

## Background

Globally, the elderly population is rapidly increasing. In 2015, based on a report released by the United Nations, 12 % of the global population was elderly individuals aged 60 years or over (United Nation, Department of Economic and Social Affairs 2015). The National Institute of Aging reported that increasing numbers of elderly individuals is associated with increases in life expectancy in most countries, including developing countries (National Institute of Aging 2011). For instance, Japan has one of the highest life expectancies, with Japanese women living to an average of 87 years old (World Health Organization 2016). Increases in the elderly population and life expectancy are related to quality of life (Demura et al. 2012; Roppolo et al. 2012), which is influenced by many factors, such as physical activity, environmental factors, social interaction, and demographics (Drewnoski and Evans 2001; Pernambuco et al. 2012; Sun et al. 2015; Heesch et al. 2015; Gao and Li 2016).

Gait, the act of walking or running in humans, is a fundamental daily physical activity (Kerrigan et al. 1998; Perry and Burnfield 2010; Baker 2013). However, advanced age can result in locomotive difficulty; specifically, with respect to walking, which is the most common of all human movements and is greatly affected by age (Winter 1991; Whittle 2007; Callisaya et al. 2008, 2010). Furthermore, walking is influenced by sex and physical condition (Whittle 2007). Accordingly, assessment of gait characteristics is essential to prevent deterioration of walking ability with advanced age. Numerous studies have addressed the changes in gait characteristics of the elderly (Watelain et al. 2000; Arif et al. 2002; Callisaya et al. 2008; Lord et al. 2013). For example, Winter et al. (1990) reported that gait characteristics are involved in a reduction in walking speed, due to a shorter stride length. Similarly, shorter step lengths and a progressive limiting of the range of motion at the ankle have been observed in the elderly (Hageman and Blanke 1986). Changes in gait characteristics of the elderly can also be identified in joint movement angles (Savelberg and Meijer 2004; Kimura et al. 2007; Kirkwood et al. 2007).

In general, a reduction in muscle strength and lower extremity function, impaired mobility, and lower activity levels by age 70 have been well documented (Guralnik et al. 1995; Winegard et al. 1996; Drewnoski and Evans 2001; Silder et al. 2008). Many previous studies have investigated age-related changes in gait variability. The majority of these studies have noted comparable gait characteristics between younger and older study groups (Mills and Barrett 2001; Grabiner et al. 2001; Moyer et al. 2006; Kang and Dingwell 2008; Schulz 2012; Vieira et al. 2015). A number of studies have previously investigated age-related changes in very elderly individuals; however, analysis of gait parameters has been limited (Kirkwood et al. 2007; Callisaya et al. 2010). In the present study, elderly Japanese women were classified into 2 subcategories, elderly and very elderly, to provide insight into whether age-related changes in gait characteristics are consistent and continued with advanced age. Moreover, it is necessary to identify gait parameters that are more reliable indicators of change in walking motions with advanced age. Spatial and temporal parameters are insufficient for a rigorous understanding of gait characteristics in elderly individuals. Despite growing agreement regarding the existence of age-related changes in gait characteristics, there is a lack of understanding regarding the changes themselves and appropriate parameters for evaluating these changes. When walking is observed using 3D motion analysis, many different parameters can be assessed. In the present study, timing-related parameters for evaluating age-related changes in gait are proposed; namely, the timing of peak joint angle and angular velocity values throughout the gait cycle. To improve the assessment of walking, it is necessary to identify parameters strongly associated with the walking motion in elderly.

In this study, basic gait parameters, gait cycle parameters, joints angle parameters, and angular velocity parameters were evaluated. Basic gait parameters included walking speed, step/stride length, and cadence, which are regarded as the most frequently used time-distance parameters for evaluating gait and walking patterns (Oberg et al. 1993; Whittle 2007; Thakurta et al. 2016). To obtain a better understanding of gait motion in elderly Japanese women, with respect to joint angle and angular velocity parameters, the peak joint angle and timing thereof were also obtained. The primary purpose of this study was to investigate the effect of aging on gait parameters in elderly Japanese women. The second purpose was to identify parameters that are reflective of gait characteristics in the elderly population. We hypothesized that differences in gait would exist among elderly and very elderly women.

## Methods

### Participants

Data were collected from 49 healthy elderly Japanese women, who were divided into 2 groups: (1) 30 elderly women (65–74.9 years old) with a mean age of 70.4 ± 2.4 years, and (2) 19 very elderly women (≥75 years old) with a mean age of 78.4 ± 2.7 years. In this study, all participants were right-leg dominant. General study group characteristics are shown in Table 1.

**Table 1:** Participant characteristics

	Group 1 (elderly women)	Group 2 (very elderly women)	*p* value
Age (years)	70.4 ± 2.4	78.4 ± 2.7	<0.01
Height (cm)	152.7 ± 5.6	148.4 ± 4.8	<0.01
Weight (kg)	50.8 ± 8.5	47.9 ± 7.4	NS
Lower-limb length (cm)	69.5 ± 3.6	66.9 ± 2.8	<0.05

All values are presented as mean ± standard deviation, unless otherwise stated

*NS* not significant

All participants were screened prior to the start of the study using a medical questionnaire. The purpose of screening evaluation was to ensure that participants did not have any serious orthopaedic symptoms in the lower-limb, and were capable of independent walking. This study was approved by the Ethics Committee, Faculty of Design, Kyushu University, and written consent was obtained prior to study procedures.

### Procedures

A 3D motion analysis system, consisting of 9 infrared cameras and Cortex software (Motion Analysis Corporation, Santa Rosa, CA, USA), was used to collect kinematic data. Participants were required to wear clothes in firm contact with the skin. Thirty-one trajectory markers were attached to the main bodily segments of each participant, including the head, upper limb (shoulder, elbow, wrist, spinal column, and sacrum), leg along the superior iliac spine, greater trochanter, lateral joint line of the knee, lateral malleolus, and toe.

### Measurements

Participants were asked to walk barefoot on a flat surface for 10 m at a self-selected speed. Prior to any measurements, participants were also instructed to practice walking barefoot. A self-selected speed measurement was recorded for each participant with the aim of defining a representative gait pattern, as performed in daily life. In this study, 3 measurements were recorded to eliminate variation in walking speed and to enhance the reliability and accuracy of the average taken from these values. Additionally, as reported by Sadeghi et al. (2002), Ko et al. (2010), and Singer et al. (2013), 3 trials are recommended for gait measurement, as a means of obtaining the most natural stride and gait patterns for each individual.

### Gait parameters

All gait parameters were analysed using KineAnalyzer software (Kissei Comtec, Nagano, Japan). In total, 70 gait parameters were measured in this study: 10 basic gait parameters (walking speed, stride length, right and left step lengths, step length, difference between right and left step lengths, ratio of step length to height, ratio of step length to lower-limb length, cadence, and walk ratio), 17 gait cycle parameters (duration of right and left swing phases, duration of right and left stance phases, duration of 1 right and left gait cycle, duration of swing phase, duration of stance phase, duration of 1 gait cycle, right and left swing phase percentages, right and left stance phase percentages, swing phase percentage, stance phase percentage, single support percentage, and double support percentage), joint angle parameters, and angular velocity parameters.

Furthermore, 21 joint angle parameters at the hip, knee, and ankle joints were analysed. At the hip and knee joints, the peak extension and flexion angles, in combination with timing of peak values and the maximal angle range, were identified. At the ankle joint, the peak plantarflexion and dorsiflexion angles, in combination with timing of peak values and the maximal angle range, were identified. Additionally, 22 angular velocity parameters were analysed. The peak extension and flexion angular velocities and timing of peak values were identified at the hip joint and knee joint, whilst the same parameters were identified for peak plantarflexion and dorsiflexion at the ankle joint.

Walking speed was calculated using step length multiplied by cadence. Stride length was measured between consecutive heel strikes of the same foot. Right step length and left step length were measured as the distance between the point of initial contact of 1 foot and the point of initial contact of the opposite foot. Step length was calculated as the average of right and left step lengths. Subsequently, the ratios of step length to height and step length to lower-limb length were derived. Cadence was defined as the number of steps taken in a given time, and walk ratio was calculated as the ratio of step length to cadence.

Phases in the gait cycle were evaluated according to duration. The duration of the right swing phase was defined as the time when the right foot was off the ground, while the right stance phase was defined as the time when the right foot was on the ground. Similarly, the duration of the left swing phase was defined as the time when the left foot was off the ground, while the left stance phase was the time when the left foot was on the ground. The single support phase was defined as the period of time during which the opposite foot was lifted for swing phase, whilst the double support phase was defined as the period when both feet were in contact with the floor. The gait cycle was defined as the time interval between 2 successive occurrences of 1 repetitive event of walking.

The percentage of total duration attributable to the swing phase was calculated using the duration of the swing phase and gait cycle. The same calculation was performed to determine the percentage of total duration attributable to the stance, single support, and double support phases, using the corresponding phase durations.

Joint angle parameters were also obtained. The angle at the hip joint was measured from 2 points on the body segment (greater trochanter and knee) with reference to the horizontal plane. Similarly, the knee joint was measured from 3 points on the body segment; specifically, the greater trochanter, knee, and lateral malleolus. Finally, the ankle joint was measured from 3 points on the body segment, including the knee, lateral malleolus, and toe.

**Fig. 1:**
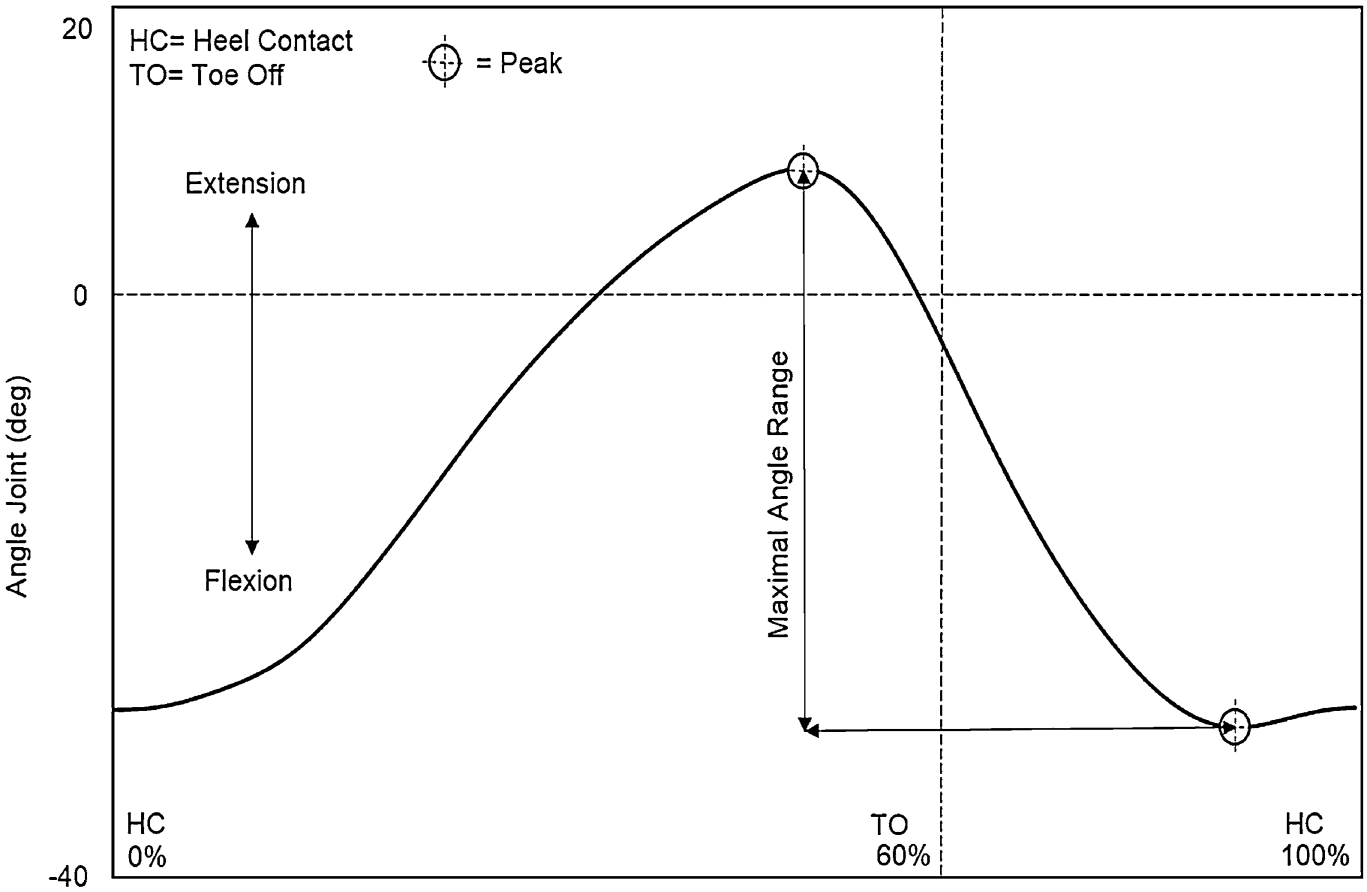
Hip joint angle

With respect to the hip joint angle, a positive value was taken to indicate hip extension, while a negative value indicated flexion. The peak extension angle was detected during the pre-swing phase, and the peak flexion angle was detected during the mid-swing phase; the maximal angle range was calculated between these maximal values (Fig. 1).

**Fig. 2:**
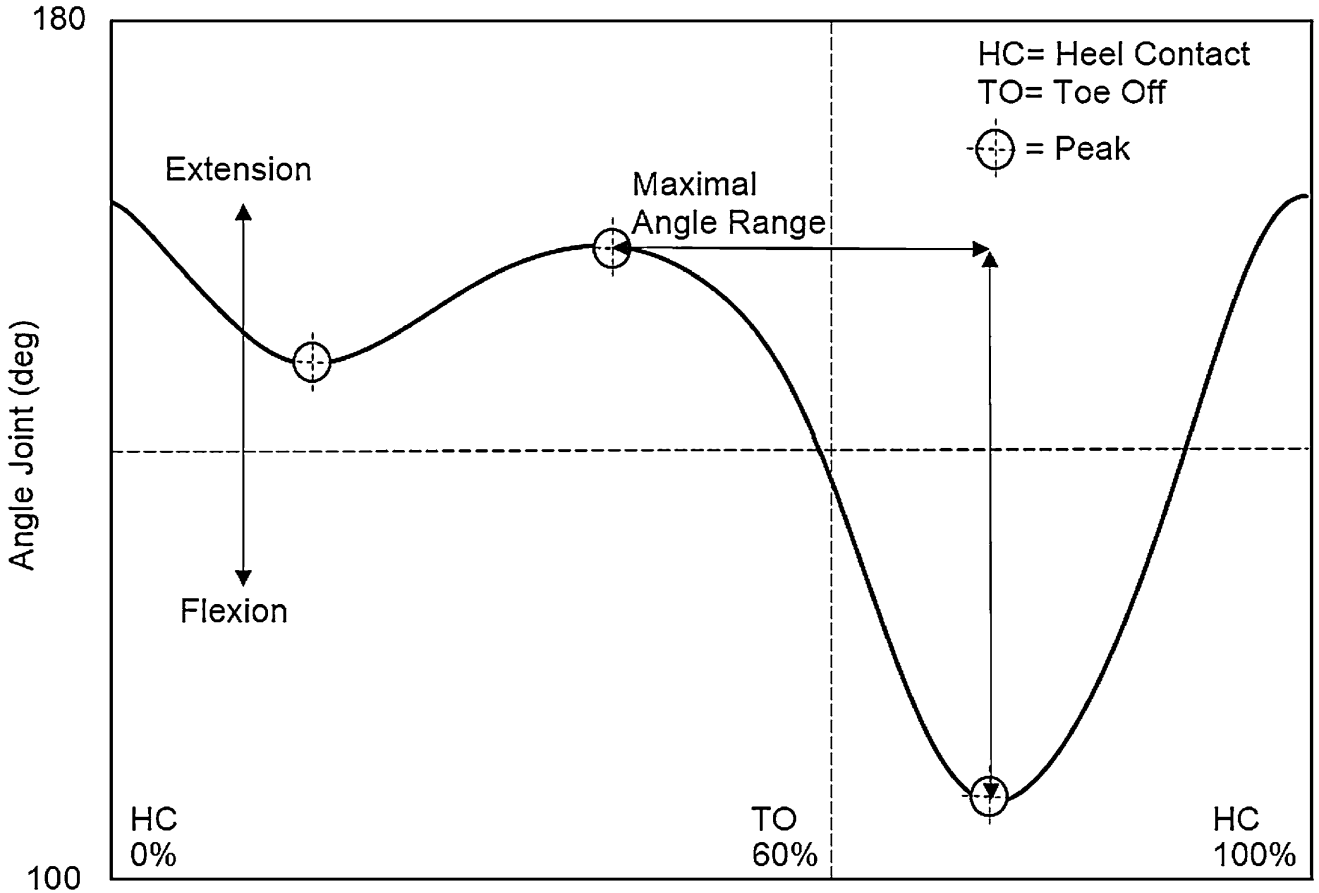
Knee joint angle

At the knee joint, an increase in angle indicated extension of the knee, while a decrease was taken to indicate knee flexion. The peak extension angle was detected during the terminal stance phase, the first peak flexion angle during the mid-stance phase, and the second peak flexion angle during initial swing phase; the maximal angle range was calculated from the peak extension angle to the second peak flexion angle (Fig. 2).

**Fig. 3:**
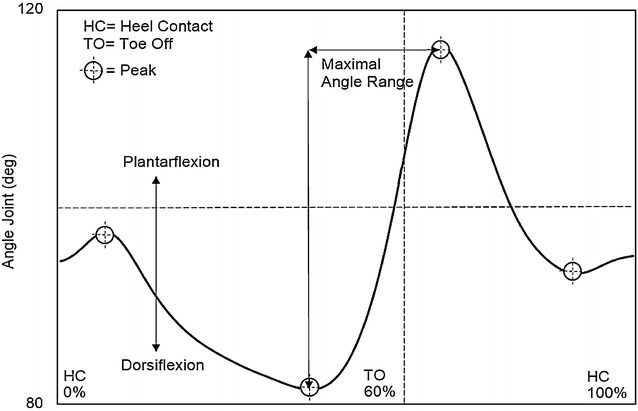
Ankle joint angle

At the ankle joint, an increased angle indicated plantarflexion, while a decrease was taken to indicate dorsiflexion. The first peak plantarflexion angle was detected during the loading response phase, the second peak plantarflexion angle during the initial swing phase, the first peak dorsiflexion angle during the terminal stance phase, and the second peak dorsiflexion angle during the mid-swing phase. The maximal angle range was calculated from the second peak plantarflexion angle to the first peak dorsiflexion angle (Fig. 3).

**Fig. 4:**
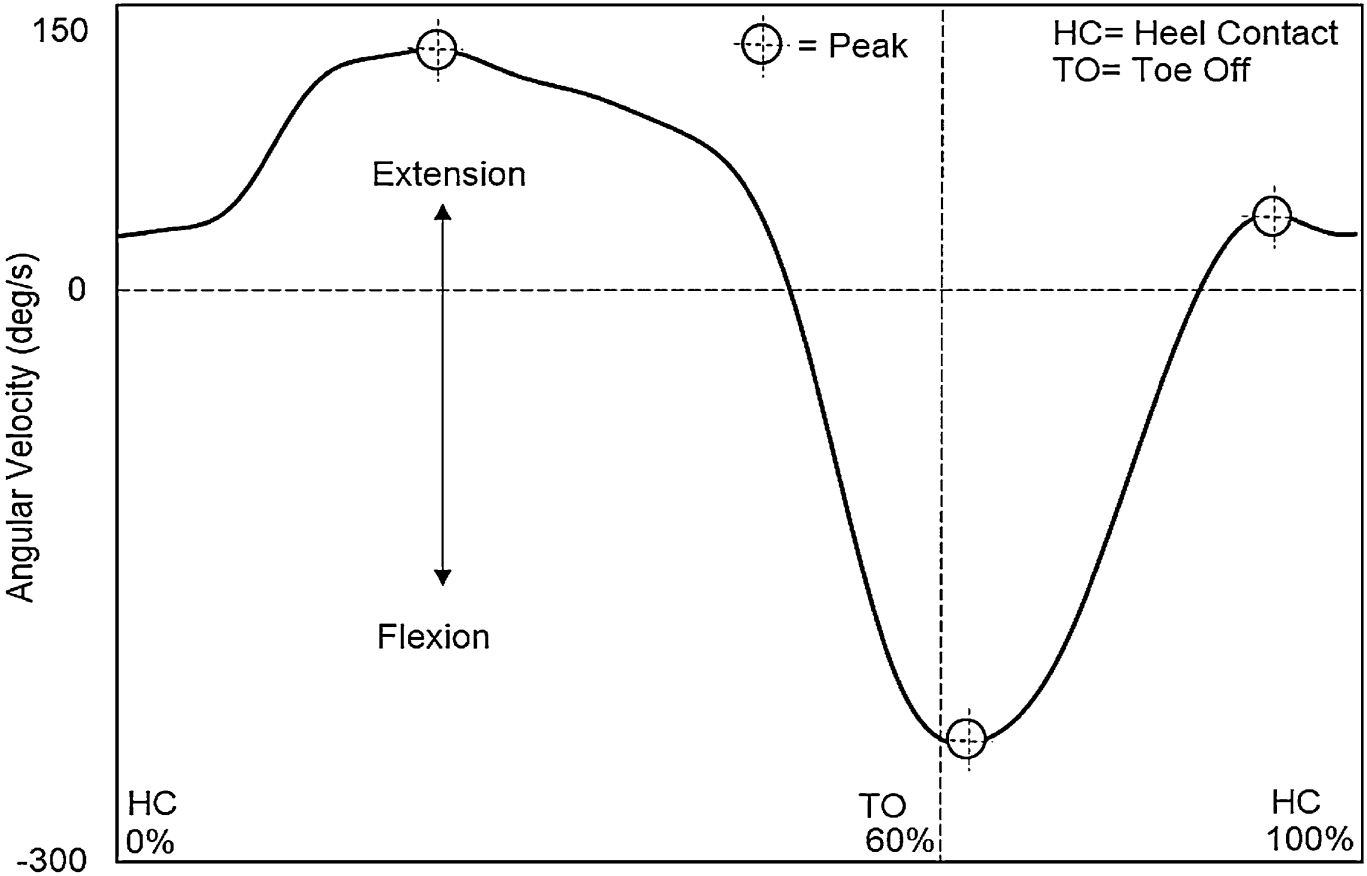
Hip angular velocity

For angular velocity parameters at the hip joint, a positive value indicated extension, while a negative value indicated flexion. The first peak extension angular velocity, second peak extension angular velocity, and peak flexion angular velocity were detected during the mid-stance phase, terminal swing phase, and initial swing phase, respectively (Fig. 4).

**Fig. 5:**
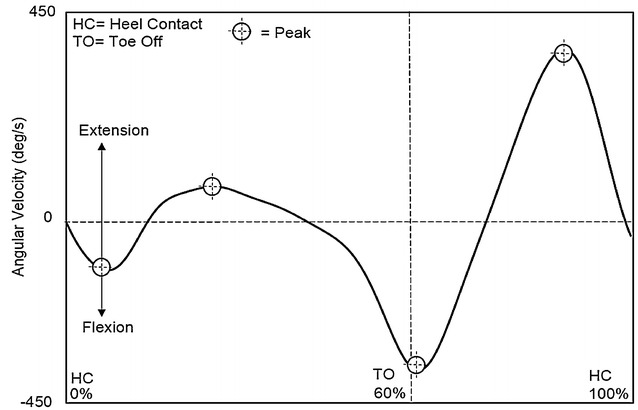
Knee angular velocity

For angular velocity parameters at the knee joint, a positive value indicated extension, while a negative value indicated flexion. The first peak extension angular velocity, second peak extension angular velocity, first peak flexion angular velocity, and second peak flexion angular velocity were detected during the mid-stance phase, terminal swing phase, loading response phase, and initial swing phase, respectively (Fig. 5).

**Fig. 6:**
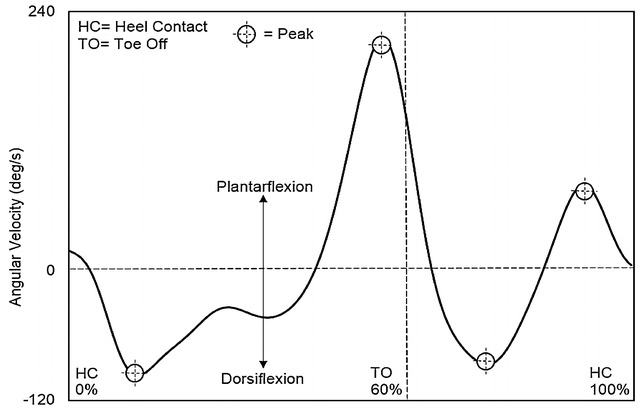
Ankle angular velocity

Finally, at the ankle joint, a positive angular velocity value indicated plantarflexion, while a negative value indicated dorsiflexion. The first peak plantarflexion angular velocity, second peak plantarflexion angular velocity, first peak dorsiflexion angular velocity, and second peak angular velocity were detected during the pre-swing phase, terminal swing phase, loading response phase, and initial swing phase, respectively (Fig. 6).

### Statistical analysis

Statistical analysis was performed using IBM SPSS Version 21.0 (Chicago, USA). Descriptive results were presented as means and standard deviation. Unpaired *t*-tests were performed to analyse differences in participant characteristics and gait parameters between elderly and very elderly Japanese women. The effect size (Cohen’s d) of parameters was calculated. The level of significance was set at 0.05.

## Results

### Participants

In the present study, the age of the very elderly women (Group 2) was significantly greater than that of the elderly women (Group 1) (*p* < 0.01). Furthermore, height and lower-limb length in Group 2 were significantly smaller than those in Group 1 (*p* < 0.01, *p* < 0.05, respectively). However, no significant difference was found in body weight between the groups (Table 1).

### Basic gait parameters

Basic gait parameters for the 2 groups are presented in Table 2. Group 2 exhibited a significantly slower walking speed (*p* < 0.05) and shorter stride length, right and left step lengths, and step length (all *p* < 0.01), than those observed in Group 1. Furthermore, the walk ratio in Group 2 was also significantly less than that of Group 1 (*p* < 0.05). However, no significant differences were found in the difference between right and step lengths, ratio of step length to height, ratio of step length to lower-limb length, or cadence.

**Table 2:** Comparison of basic gait parameters between elderly women and very elderly women

Parameters	Group 1 (elderly women)	Group 2 (very elderly women)	*p* value	Effect size
Walking speed (m/min)	75.3 ± 10.0	68.8 ± 8.1	<0.05	0.71
Stride length (cm)	122.0 ± 11.5	112.5 ± 7.5	<0.01	0.97
Right step length (cm)	60.6 ± 5.9	55.8 ± 3.8	<0.01	0.97
Left step length (cm)	61.1 ± 5.9	56.1 ± 3.8	<0.01	1.01
Step length (cm)	60.9 ± 5.7	55.9 ± 3.6	<0.01	1.04
Difference between right and left step lengths (cm)	2.5 ± 1.7	2.0 ± 1.5	NS	0.31
Ratio of step length to height	0.40 ± 0.04	0.38 ± 0.03	NS	0.56
Ratio of step length to lower-limb length	0.88 ± 0.08	0.84 ± 0.08	NS	0.50
Cadence (steps/min)	123.6 ± 10.5	122.5 ± 8.7	NS	0.11
Walk ratio	0.50 ± 0.06	0.46 ± 0.03	<0.05	0.84

All values are presented as mean ± standard deviation, unless otherwise stated

*NS* not significant

### Gait cycle parameters

A comparison of gait cycle parameters between the 2 groups is presented in Table 3. Significant differences were observed in the percentage of total duration attributable to the swing and stance phases (*p* < 0.05). The swing phase percentage in Group 2 was significantly smaller than that of Group 1 (*p* < 0.05). Conversely, the stance phase percentage in Group 2 was significantly greater than that of Group 1 (*p* < 0.05). However, there were no significant differences observed between the groups with respect to the durations of the swing phase, stance phase, duration of gait cycle, single support percentage, or double support percentage.

**Table 3:** Comparison of gait cycle parameters between elderly women and very elderly women

Parameters	Group 1 (elderly women)	Group 2 (very elderly women)	*p* value	Effect size
Duration of right swing phase (s)	0.38 ± 0.03	0.38 ± 0.02	NS	0.00
Duration of right stance phase (s)	0.59 ± 0.06	0.61 ± 0.05	NS	0.36
Duration of right gait cycle (s)	0.98 ± 0.08	0.98 ± 0.07	NS	0.00
Duration of left swing phase (s)	0.38 ± 0.03	0.38 ± 0.02	NS	0.00
Duration of left stance phase (s)	0.59 ± 0.06	0.60 ± 0.05	NS	0.18
Duration of left gait cycle (s)	0.98 ± 0.08	0.99 ± 0.07	NS	0.13
Duration of swing phase (s)	0.38 ± 0.02	0.38 ± 0.02	NS	0.00
Duration of stance phase (s)	0.59 ± 0.06	0.61 ± 0.05	NS	0.36
Duration of 1 gait cycle (s)	0.98 ± 0.08	0.99 ± 0.07	NS	0.18
Right swing phase percentage (%)	39.3 ± 1.5	38.5 ± 1.3	NS	0.56
Right stance phase percentage (%)	60.7 ± 1.5	61.5 ± 1.3	NS	0.57
Left swing phase percentage (%)	39.4 ± 1.3	38.7 ± 1.2	NS	0.56
Left stance phase percentage (%)	60.7 ± 1.3	61.3 ± 1.2	NS	0.48
Swing phase percentage (%)	39.4 ± 1.3	38.6 ± 1.1	<0.05	0.66
Stance phase percentage (%)	60.6 ± 1.3	61.4 ± 1.1	<0.05	0.66
Single support percentage (%)	78.6 ± 2.6	77.2 ± 2.2	NS	0.58
Double support percentage (%)	21.4 ± 2.6	22.8 ± 2.2	NS	0.58

All values are presented as mean ± standard deviation, unless otherwise stated

*NS* not significant

### Joint angle parameters

Significant differences were observed in the peak extension timing of the hip joint, second peak flexion timing of the knee joint, and second peak plantarflexion timing of the ankle joint (*p* < 0.05, *p* < 0.01, and *p* < 0.05, respectively).

Table 4 shows the results of all joint angle parameters. At the hip joint, the peak extension timing in Group 2 was significantly later than that observed in Group 1 (*p* < 0.05). This observation was also true at the knee and ankle joints, with the second peak flexion (*p* < 0.01) and second peak plantarflexion (*p* < 0.05), respectively, occurring later in Group 2 than in Group 1. However, no other significant differences were noted with respect to joint angle parameters.

**Table 4:** Comparison of joint angle parameters between elderly women and very elderly women

Parameters	Group 1 (elderly women)	Group 2 (very elderly women)	*p* value	Effect size
Hip joint				
Peak extension angle (°)	19.4 ± 4.0	18.4 ± 4.8	NS	0.22
Peak extension timing (%)	54.2 ± 1.2	54.9 ± 0.7	<0.05	0.71
Peak flexion angle (°)	−27.0 ± 2.6	−27.8 ± 2.4	NS	0.32
Peak flexion timing (%)	88.1 ± 1.8	88.9 ± 1.6	NS	0.47
Maximal angle range (°)	46.4 ± 3.8	46.3 ± 4.4	NS	0.02
Knee joint				
Peak extension angle (°)	172.0 ± 5.4	170.1 ± 5.8	NS	0.34
Peak extension timing (%)	40.8 ± 3.2	42.0 ± 3.6	NS	0.35
First peak flexion angle (°)	157.2 ± 6.0	156.6 ± 4.2	NS	0.11
First peak flexion timing (%)	13.4 ± 1.1	13.1 ± 1.8	NS	0.20
Second peak flexion angle (°)	114.4 ± 5.2	115.3 ± 4.6	NS	0.18
Second peak flexion timing (%)	73.4 ± 1.1	74.3 ± 0.9	<0.01	0.89
Maximal angle range (°)	57.5 ± 4.5	54.8 ± 4.9	NS	0.57
Ankle joint				
First peak plantarflexion angle (°)	102.1 ± 4.7	101.9 ± 3.6	NS	0.48
First peak plantarflexion timing (%)	5.9 ± 1.3	5.4 ± 1.2	NS	0.40
Second peak plantarflexion angle (°)	111.6 ± 5.7	111.4 ± 6.3	NS	0.03
Second peak plantarflexion timing (%)	63.9 ± 1.5	65.0 ± 1.5	<0.05	0.73
First peak dorsiflexion angle (°)	84.5 ± 4.3	84.4 ± 3.5	NS	0.02
First peak dorsiflexion timing (%)	41.6 ± 5.3	43.2 ± 3.5	NS	0.35
Second peak dorsiflexion angle (°)	92.0 ± 5.0	92.4 ± 3.2	NS	0.09
Second peak dorsiflexion timing (%)	83.8 ± 2.4	84.7 ± 1.6	NS	0.44
Maximal angle range (°)	27.1 ± 4.1	27.0 ± 4.2	NS	0.02

All values are presented as mean ± standard deviation, unless otherwise stated

*NS* not significant

### Angular velocity parameters

Table 5 shows the results of all angular velocity parameters. At the hip joint, the second peak extension timing and peak flexion timing in Group 2 were significantly later than those in Group 1 (*p* < 0.05). Again, this relationship held true for the knee and ankle joints; the second peak flexion timing at the knee (*p* < 0.05), and the first peak plantarflexion timing at the ankle (*p* < 0.05) were both later in Group 2 than those observed in Group 1. No significant differences were found in any other angular velocity parameters at the hip, knee, or ankle joints.

**Table 5:** Comparison of angular velocity parameters between elderly and very elderly women

Parameters	Group 1 (elderly women)	Group 2 (very elderly women)	*p* value	Effect size
Hip joint				
First peak extension angular velocity (°/s)	139.6 ± 26.2	129.5 ± 22.9	NS	0.41
First peak extension timing (%)	21.2 ± 2.9	20.3 ± 3.5	NS	0.28
Second peak extension angular velocity (°/s)	43.1 ± 21.8	41.5 ± 17.2	NS	0.08
Second peak extension timing (%)	93.2 ± 2.7	94.7 ± 2.1	<0.05	0.62
Peak flexion velocity (°/s)	−232.7 ± 29.5	−226.8 ± 39.7	NS	0.17
Peak flexion timing (%)	66.8 ± 1.6	67.8 ± 1.5	<0.05	0.64
Knee joint				
First peak extension angular velocity (°/s)	95.3 ± 27.5	87.0 ± 29.0	NS	0.29
First peak extension timing (%)	23.9 ± 3.1	23.1 ± 3.9	NS	0.23
Second peak extension angular velocity (°/s)	403.6 ± 50.7	379.2 ± 55.4	NS	0.46
Second peak extension timing (%)	88.7 ± 1.1	89.0 ± 1.3	NS	0.25
First peak flexion angular velocity (°/s)	−176.8 ± 47.7	−157.1 ± 38.6	NS	0.45
First peak flexion timing (%)	5.9 ± 1.3	6.0 ± 1.3	NS	0.07
Second peak flexion angular velocity (°/s)	−400.1 ± 50.7	−378.0 ± 52.9	NS	0.42
Second peak flexion timing (%)	62.3 ± 1.1	63.2 ± 1.6	<0.05	0.65
Ankle joint				
First peak plantarflexion angular velocity (°/s)	275.2 ± 43.8	264.9 ± 44.9	NS	0.23
First peak plantarflexion timing (%)	57.3 ± 2.1	58.5 ± 1.5	<0.05	0.65
Second peak plantarflexion angular velocity (°/s)	81.7 ± 23.8	87.7 ± 33.8	NS	0.66
Second peak plantarflexion timing (%)	91.8 ± 2.6	92.4 ± 2.7	NS	0.22
First peak dorsiflexion angular velocity (°/s)	−111.1 ± 21.0	−104.4 ± 20.7	NS	0.32
First peak dorsiflexion timing (%)	12.0 ± 1.9	11.3 ± 1.6	NS	0.40
Second peak dorsiflexion angular velocity (°/s)	−167.3 ± 41.9	−161.2 ± 52.0	NS	0.13
Second peak dorsiflexion timing (%)	71.3 ± 2.9	72.5 ± 2.4	NS	0.45

All values are presented as mean ± standard deviation, unless otherwise stated

*NS* not significant

## Discussion

### Basic gait parameters

The key finding of this study is that a number of gait parameters are strongly associated with aging, as demonstrated in Japanese women across 2 subcategories (elderly and very elderly) in the 65–84 years age range. Slower walking speeds, shorter stride lengths, shorter step lengths, and a smaller walk ratio were observed in the very elderly age group, when compared with elderly participants. Slower walking speeds in the elderly have been reported in many studies (Himann et al. 1988; Kaneko et al. 1991; Bohannon 1997; Prince et al. 1997; Callisaya et al. 2008). In this study, a decrease in the walking speed of the very elderly was primarily the product of a decrease in step/stride length; this observation has also been made in a number of previous studies (Bohannon 1997; Schulz 2012; Afiah et al. 2014). Moreover, shorter step/stride length in very elderly women was largely associated with short stature. This observation is in agreement with those made by Bendall et al. (1989), Kimura et al. (2007), and Winter et al. (1990), where it was noted that height is directly related to stride length and walking speed. Furthermore, very elderly women showed smaller walk ratios than their elderly counterparts. The walk ratio is a speed-independent indicator of walking patterns, and is derived from step length and step-rate (Sekiya and Nagasaki 1998). Crosbie et al. (1997) found that gait variability in older adults is more likely to be step length-dependent, due to a limited gait capacity when compared to younger individuals. Therefore, the present study serves to extend observations made previously, in that smaller walk ratios in very elderly women are indicative of a change in gait behaviour from a step length-dependent to cadence-dependent pattern; however, no significant differences were found with respect to cadence.

Furthermore, no significant differences were found in cadence between elderly and very elderly women. This finding is compatible with a number of previous studies, which reported that elderly individuals maintain a normal cadence during walking (Winter 1991; Watelain et al. 2000; Silder et al. 2008). Such observations are thought to be a product of generally high activity levels in elderly participants, and are aided by the absence of gait-related pathologies (Winter 1991). The elderly participants included in this study were also moderately active and had a reasonably good walking ability; therefore, it may be difficult to observe any significant difference with respect to cadence between elderly and very elderly women.

### Gait cycle parameters

Importantly, when analysing the complete gait cycle, it was also observed that very elderly women exhibit a smaller percentage of swing phase and greater percentage of stance phase, when compared with the elderly group. The swing phase is characterised by progression of the swing limb between consecutive support positions, and constitutes the basis of forward movement (Winter et al. 1990; Mills and Barrett 2001). The swing phase begins immediately after toe-off, and is determined by swing limb advancement. The stance phase involves a period of bilateral foot contact with the floor (double stance phase) at the beginning and end of a gait cycle (Perry and Burnfield 2010). In the present study, the smaller percentages of swing phase noted in very elderly women may be a product of reductions in swing limb advancement and muscle power, while greater percentages of stance phase might be caused by diminished limb and trunk stability. These results are similar to those reported by Whittle (2007) and Perry and Burnfield (2010), whereby normal swing acceleration is decreased due to hip flexor weakness and slow limb advancement in the elderly. An increase in the duration of the stance phase was taken to indicate difficulty with maintaining balance while walking in elderly people. In addition, a greater percentage of stance phase suggests that extra time after completion of limb advancement is needed for elderly women to maintain weight-bearing stability; however, it should be noted that no significant difference was found in the percentage duration of the double support phase. This result is analogous to that observed by Kimura et al. (2007), who found that a longer stance phase is a primary characteristic of the elderly, as a response to impaired balance during walking.

### Joint angle parameters

It is well established that a number of joint angle movements are characteristic of the gait patterns observed in the elderly (Winter et al. 1990; Woollacott and Tang 1997; Kirkwood et al. 2007; Schmitz et al. 2009). The present study provides novel extension to those performed previously, by analysing essential joint angles at the hip, knee and ankle; specifically, the timing of peak angle throughout the gait cycle. In this study, both the peak angle and the temporal relationship of this value with the complete gait cycle were analysed as a means of improving understanding of gait characteristics in elderly Japanese women. Interestingly, the only significant differences noted between elderly and very elderly women were related to the timing of peak joint angles; namely, the peak extension timing at the hip joint, peak extension timing at the knee joint, and second peak plantarflexion timing at the ankle joint.

No significant differences were found in the peak values of any joint angle between elderly and very elderly women, in the present study. This finding is discordant with a number of previous studies regarding the effect of aging on joint angle parameters. For instance, DeVita and Hortobagyi (2000) reported that joint angular kinematics at the hip, knee, and ankle joints significantly differed according to age. Similarly, Ko et al. (2010) identified decreases in range of motion at the hip and ankle joints, as well as a reduction in peak plantarflexion angle in elderly people. In contrast, a study from Mills and Barrett (2001) reported no differences in the peak value of any joint angle, and suggested that this may be a product of differences in gait speed or stride length, general health condition, and activity levels in elderly participants. In the present study, the lack of significant differences in joint angle measurements is likely due to consistency in cadence and the ratio of step length to lower-limb length between elderly and very elderly women. Moreover, previous differences in peak joint angle measurements have primarily been noted when comparing young and elderly age groups, rather than subsets of the elderly (Judge et al. 1996; Prince et al. 1997; Kerrigan et al. 1998; DeVita and Hortobagyi 2000). For instance, Kerrigan et al. (1998) pointed out that during comfortable walking, significant reductions were found in peak hip extension. Judge et al. (1996) also reported that older adults exhibit reduced peak ankle plantarflexion during the late stance phase, which constituted the primary contributing factor to shorter step lengths in the elderly, when compared to young participants.

When analysing the timing of peak joint angles, it was noted that delayed peak timing begun at approximately 54 % of the gait cycle. At the hip joint, peak extension timing in very elderly women was delayed when compared to elderly women, and occurred during the pre-swing phase (50–62 % of the gait cycle) when the hip joint transferred from a flexed to extended position. In this phase, terminal double limb support is initiated by floor contact of the contralateral limb (Perry and Burnfield 2010). A number of previous studies have identified gait characteristics of the hip joint in elderly individuals (Kerrigan et al. 1998; DeVita and Hortobagyi 2000; Schmitz et al. 2009; Anderson and Madigan 2014). For instance, Alcock et al. (2013) suggested that an extra increase in power generation at the hip is required during the pre-swing phase in elderly adults, due to a reduction in gait capacity. In addition, Kerrigan et al. (1998) reported that a reduction in peak hip extension angle during walking is associated with an increase in anterior pelvic tilt and a reduced range of motion in hip extension; these factors, prevalent in elderly individuals, may contribute to age-related differences in gait motion (Anderson and Madigan 2014). In keeping with studies performed by Kerrigan et al. (1998), Alcock et al. (2013), and Anderson and Madigan (2014), the delay in peak extension timing of the hip joint in very elderly women might be a reflection of several factors, such as the extra effort required to change the hip joint position, poor hip flexor power for push-off, and a reduction in the range of extension.

Delayed peak joint angle timing was also detected during the initial swing phase (62–75 % of gait cycle). In this phase, delayed timing was noted when the knee and ankle joints transferred from extended to flexed and dorsiflexed to plantarflexed positions, respectively. The initial swing phase begins when the foot leaves the ground and continues until maximum knee flexion occurs (Perry and Burnfield 2010). Knee flexion is essential in lifting the foot for swing limb advancement and to facilitate foot clearance during the initial swing phase (Nene et al. 1999). Indeed, diminished knee flexion during the initial swing phase is associated with knee stiffness during walking (Goldberg et al. 2003). Moreover, elderly individuals appear to produce less isokinetic ankle plantarflexion during walking (Silder et al. 2008), have a more limited range of motion at the knee joint (Kirkwood et al. 2007), and exhibit poorer propulsion during the push-off period as a product of smaller plantarflexor torques (Cho et al. 2004). In addition, Ko et al. (2011) reported that elderly women exhibit poorer capacity to advance the swing limb forwards during the swing phase, as a product of less generative mechanical work expenditure from the knee joint. In a report from Shin et al. (2012), there was a significant correlation between muscle strength and gait variability at the knee joint. Accordingly, it can be suggested that the delayed peak angle timings observed during the initial swing phase at the knee and ankle joints are a product of knee stiffness, a reduction in push-off torque, less power to lift the limb, and deteriorating muscle strength with age.

**Fig. 7:**
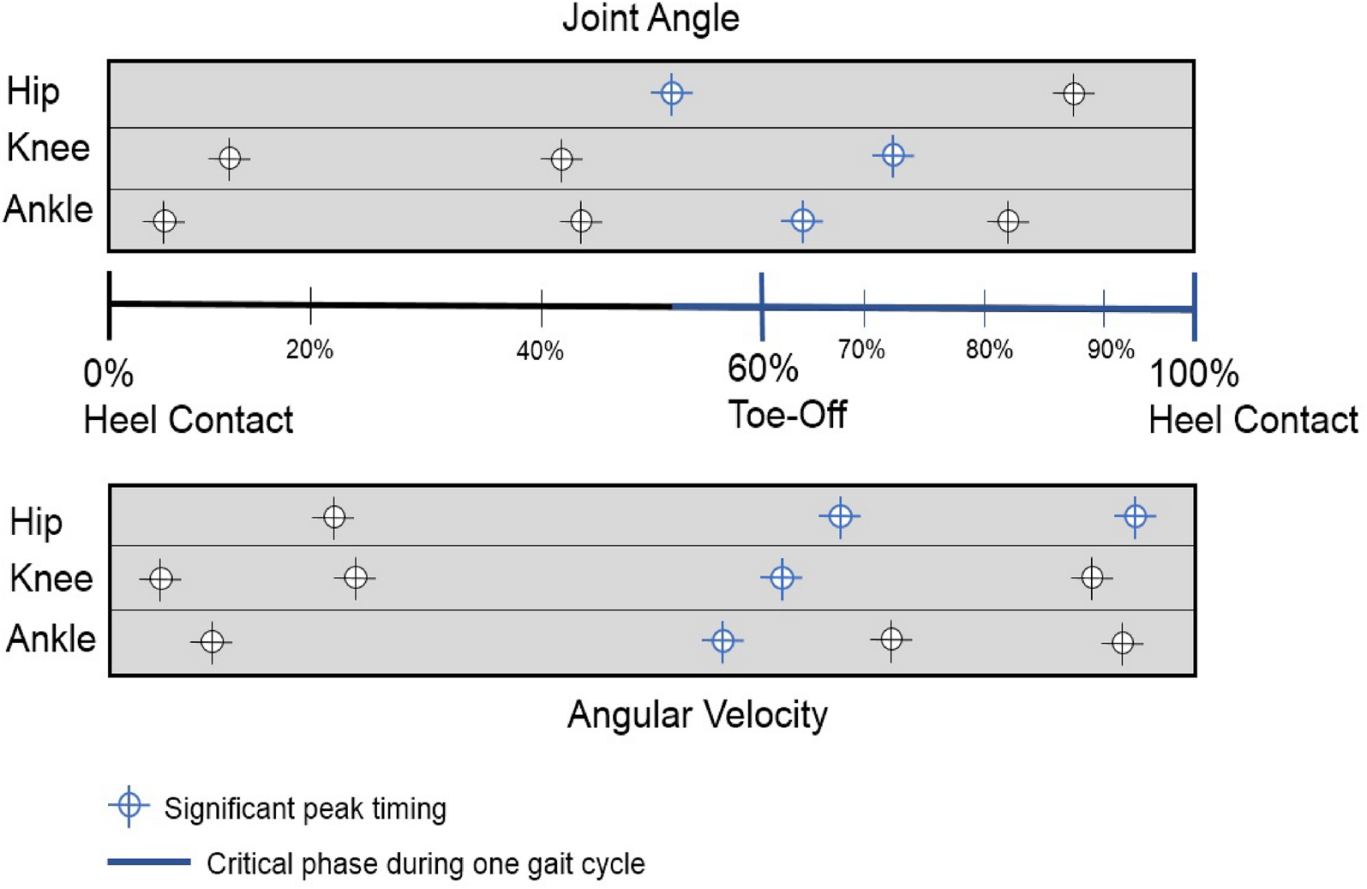
The peak timing of joint angle and angular velocity parameters

It is also possible that delayed peak angle timings are affected by delays during the previous gait phase. For instance, the first delay in angular timing is observed at approximately 54 % of the gait cycle; consequently, this may then affect peak angle timing in the subsequent phases (Fig. 7). However, delayed peak timing does not appear to continue from 76 % until the end of the gait cycle. Accordingly, delays in peak angle timing are likely to primarily reflect unique joint behaviour in the very elderly.

### Angular velocity parameters

To our knowledge, this study is the first to provide a comparison of elderly and very elderly women with respect to peak angular velocities, and the temporal relationship of these peak values to the gait cycle, at the hip, knee, and ankle joints. However, significant differences were only noted in the timing of peak values throughout the gait cycle, rather than the peak velocities themselves. Peak values occurred during the pre-swing phase, initial swing phase, and terminal swing phase. Previously identified significant differences in peak angular velocity parameters have primarily been observed in comparison with younger (Watelain et al. 2000; Mills and Barrett 2001; Anderson and Madigan 2014) and middle-age participants (Ko et al. 2012). If the timing of the peak joint angle parameter is representative of the timing of joint angle displacement, then the timing of the peak angular velocity is representative of the greatest moment of instantaneous change in flexion/extension. Therefore, in combination with the timing of peak joint angle movements, angular velocity is necessary to obtain a better understanding of gait characteristics in the elderly.

Delayed peak angular velocity begun in the pre-swing phase (52–62 % of gait cycle), and was subsequently noted in the initial swing phase (62–75 % of gait cycle) and terminal swing phase (87–100 % of gait cycle). At the hip joint, the timing of the second peak extension in very elderly women occurred during the terminal swing phase (87–100 % of gait cycle), which represents a delay when compared with elderly women. This corresponds to the point at which the hip joint is changing from a flexed to extended position. This phase is characterised by the deceleration of the swinging limb due to hip extensor activity (Rancho Los Amigos National Rehabilitation Center 1989). During this phase, the hip maintains its earlier flexion (Kharb et al. 2011), while muscle action prepares the limb for stance by stopping further flexion (Perry and Burnfield 2010). During the terminal swing phase, limb retraction improves shock attenuation as a strategy to enhance foot ground-speed matching (Endo et al. 2014), and hamstring muscles rapidly increase the intensity of their action (Perry and Burnfield 2010). At the end of the terminal swing phase, however, activity of the hamstrings decreases, and the limb is optimally positioned for initial contact (Perry and Burnfield 2010). Optimising the initial limb contact plays an important role in shock absorption. In the elderly, stride length is shortened as a means to compensate for a reduction in shock absorption capacity at the knee (Watelain et al. 2000). In addition, delayed and reduced hamstring muscle activation in the elderly is affected by the heel contact velocity (Prince et al. 1997; Lockhart and Kim 2006). Therefore, it is suggested that a delay in the timing of the peak hip angular velocity is related to a reduced necessity for shock absorption, due to a shorter stride length and/or slower adjustment of muscle activity at the hamstrings for shock absorption before heel contact in very elderly women.

Overall, findings in this study support the hypothesis that differences in gait appear between the subcategories of elderly and very elderly in Japanese women. Furthermore, a number of differences were noted in the timing of peak angle and angular velocity parameters between elderly and very elderly women. Interestingly, these peak timings appeared in the pre-swing, initial swing, and terminal swing phases (Fig. 7), which are related to the position and advancement of the limb from its trailing position, toe clearance, and preparation for the next initial contact, respectively. Therefore, understanding joint angle and angular velocity parameters is essential to develop an understanding of aging-related changes in gait.

### Implications and limitations

To our knowledge, gait parameters related to the peak timing of each joint angle movement and angular velocity have not previously been examined. Therefore, these findings may be useful as a means of identifying and monitoring parameters that are reflective of walking ability in the elderly population. A limitation of the present study was the inclusion of only elderly subcategories, rather than a young or middle-aged comparison group. Furthermore, the peak value and timing of angular acceleration parameters were not evaluated. Therefore, in further investigation, it would be interesting to compare the parameters noted herein with measurements of angular acceleration, and to evaluate gait differences across a wider age range.

## Conclusions

This study investigated the effect of aging on gait parameters in elderly Japanese women. A number of parameters (walking speed, stride length, step length, walk ratio, swing phase percentage, stance phase percentage, and peak timing of joints angle and angular velocity) showed significant differences between elderly and very elderly women. Our results suggest that examining peak values and the timing of joint angle and angular velocity parameters at the hip, knee, and ankle joints are the best reflection of changes in gait with age.
